# Anastomotic techniques for oesophagectomy for malignancy: systematic review and network meta‐analysis

**DOI:** 10.1002/bjs5.50298

**Published:** 2020-05-23

**Authors:** S. K. Kamarajah, J. R. Bundred, P. Singh, S. Pasquali, E. A. Griffiths

**Affiliations:** ^1^ Department of Hepatobiliary, Pancreatic and Transplant Surgery, Freeman Hospital Newcastle upon Tyne Hospitals NHS Foundation Trust Newcastle upon Tyne UK; ^2^ Institute of Cellular Medicine University of Newcastle Newcastle upon Tyne UK; ^3^ College of Medical and Dental Sciences Birmingham UK; ^4^ Institute of Cancer and Genomic Sciences, College of Medical and Dental Sciences University of Birmingham Birmingham UK; ^5^ Department of Upper Gastrointestinal Surgery University Hospitals Birmingham NHS Foundation Trust Birmingham UK; ^6^ Regional Oesophago‐Gastric Unit Royal Surrey NHS Foundation Trust Guildford UK; ^7^ Department of Surgery Fondazione IRCCS Istituto Nazionale dei Tumori Milan Italy

## Abstract

**Background:**

Current evidence on the benefits of different anastomotic techniques (hand‐sewn (HS), circular stapled (CS), triangulating stapled (TS) or linear stapled/semimechanical (LSSM) techniques) after oesophagectomy is conflicting. The aim of this study was to evaluate the evidence for the techniques for oesophagogastric anastomosis and their impact on perioperative outcomes.

**Methods:**

This was a systematic review and network meta‐analysis. PubMed, EMBASE and Cochrane Library databases were searched systematically for randomized and non‐randomized studies reporting techniques for the oesophagogastric anastomosis. Network meta‐analysis of postoperative anastomotic leaks and strictures was performed.

**Results:**

Of 4192 articles screened, 15 randomized and 22 non‐randomized studies comprising 8618 patients were included. LSSM (odds ratio (OR) 0·50, 95 per cent c.i. 0·33 to 0·74; *P* = 0·001) and CS (OR 0·68, 0·48 to 0·95; *P* = 0·027) anastomoses were associated with lower anastomotic leak rates than HS anastomoses. LSSM anastomoses were associated with lower stricture rates than HS anastomoses (OR 0·32, 0·19 to 0·54; *P* < 0·001).

**Conclusion:**

LSSM anastomoses after oesophagectomy are superior with regard to anastomotic leak and stricture rates.

## Graphical abstract

Stapled anastomoses were associated with lower anastomotic leak rates than hand‐sewn anastomoses following oesophagectomy. Furthermore, linear stapled/semimechanical (LSSM) techniques were associated with lower rates of anastomotic stricture than circular stapled or hand‐sewn anastomoses. Current evidence therefore suggests superiority for the LSSM technique for oesophagogastric anastomosis.

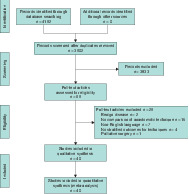

Linear stapler techniques superior

## Abstract (es)

**Antecedentes:**

La evidencia actual sobre los beneficios de diferentes técnicas de anastomosis, incluyen la técnica manual (*hand‐sewn*, HS), la sutura mecánica circular (*circular stapled*, CS), la sutura mecánica triangular (*triangular stapler*, TS) o la sutura mecánica lineal/semi‐mecánica (*linear stapler/semi‐mechanical*., LSSM) tras una esofaguectomía es contradictoria. El objetivo de este estudio fue evaluar la evidencia referente a las técnicas de anastomosis esofagogástrica (*oesophagogastric*, OG) y su impacto sobre los resultados perioperatorios.

**Métodos:**

Se efectuó una revisión sistemática y metaanálisis en red, basados en una búsqueda sistemática en las bases de datos PubMed, EMBASE y Cochrane Library de estudios aleatorizados y no aleatorizados que describiese técnicas para la anastomosis OG. Se llevó a cabo un metaanálisis en red para los resultados de fugas anastomóticas y estenosis postoperatorias.

**Resultados:**

De los 4.192 artículos revisados, se incluyeron 15 estudios aleatorizados y 22 no aleatorizados con un total de 8.618 pacientes. Las anastomosis con LSSM (razón de oportunidades, *odds ratio*, OR 0,49, i.c. del 95%: 0,33‐0,74, *P* = 0,001) y las anastomosis con CS (OR 0,68, i.c. del 95%: 0.48‐0,95, *P* = 0,027) se asociaron con tasas de fugas anastomóticas más bajas que las anastomosis con HS. Las anastomosis con LSSM se asociaron con unas tasas más bajas de estenosis (OR 0,15, i.c. del 95%: 0,08‐0,28, *P* < 0,001), frente a las anastomosis con TS y HS.

**Conclusiones:**

Las anastomosis con LSSM después de esofaguectomía son superiores en relación a las tasas de fugas anastomóticas y estenosis.

## Introduction

Despite improvements in perioperative care over recent decades, which have led to improved patient selection, reduced operative morbidity and mortality, and prolonged postoperative survival^1^, ^2^, anastomotic leak remains the most serious technical complication after oesophagectomy. Patients who experience anastomotic leakage suffer high morbidity, have a high postoperative mortality rate, ranging between 21 and 35 per cent, incur high hospital costs^3^, ^4^, ^5^, ^6^, ^7^. They also suffer long‐term effects, such as an increased risk of anastomotic stricture and poorer long‐term survival, compared with patients who recover uneventfully^8^. Many perioperative factors are thought to be responsible for anastomotic integrity after oesophagectomy, such as surgical approach, tumour location (cervical or thoracic) and technique of oesophagogastric anastomosis^9^.

Several meta‐analyses^10^, ^11^, ^12^, ^13^, ^14^ have compared stapled and hand‐sewn anastomotic techniques. These studies have included both randomized and non‐randomized trials, and have found no significant differences in anastomotic leak rates between the two anastomotic techniques. Most individual comparative studies, however, chose either to look at two types of stapled anastomosis or to group all stapled anastomoses together. There is a paucity of literature comparing all anastomotic techniques described in this study. Anastomotic techniques can include hand‐sewn (HS), circular stapled (CS), linear stapled/semimechanical (LSSM)^15^, ^16^ and triangulating stapled (TS)^17^, ^18^. There are encouraging reports of low anastomotic leak rates when linear stapled techniques are employed^19^. The strength of performing a network meta‐analysis is that it allows the evaluation of treatments that have not been compared directly (for example, comparison of B *versus* C, using data from studies comparing A *versus* B and A *versus* C). Network meta‐analysis ranks multiple treatments based on their efficacy, and pools together direct and indirect evidence within mixed comparisons, improving the precision of estimates^20^, ^21^.

The aim of this systematic review was to evaluate current evidence and perform a network meta‐analysis to identify techniques associated with superior perioperative outcomes in patients undergoing oesophagectomy for oesophageal cancer.

## Methods

This was a systematic review and network meta‐analysis. The study was registered with the PROSPERO database (Registration CRD42018106086) and reported according to the PRISMA guidelines^22^.

### Search strategy

A systematic search of PubMed, EMBASE and Cochrane Library databases was conducted by two independent investigators on 22 April 2019, to include studies up to 31 March 2019. Search terms included ‘oesophageal cancer’ or ‘esophageal cancer’ or ‘gastro‐oesophageal cancer’, and ‘anastomosis’ or ‘hand‐sewn’ or ‘linear stapler’ or ‘circular stapler’ individually or in combination (*Table* *S1*, supporting information). The ‘related articles’ function was used to broaden the search, and all citations were considered for relevance. A manual search of reference lists in recent reviews was also undertaken. After excluding duplicates, two researchers independently reviewed the titles and abstracts of studies identified by the literature search. If a study was considered to be potentially relevant to the research question, the full publication was reviewed. Reference lists of all included studies were hand‐searched to identify other potentially relevant studies. Any areas of disagreement between the two primary researchers were resolved through discussion with all authors.

### Inclusion and exclusion criteria

Inclusion criteria included studies reporting the comparison of anastomotic technique (by any method) in patients with oesophageal cancer who underwent oesophagectomy, published in the English language. Exclusion criteria included conference abstracts, review articles, case reports (fewer than 5 patients), and publications with mixed populations, in which the outcomes of patients with either benign disease or cancer at another site could not be separated from those of patients with oesophageal cancer.

### Study outcomes

The primary outcome measures were anastomotic complications, including anastomotic leak or stricture^23^. Secondary outcome measures were surgery‐specific complications (pulmonary, cardiac) and death (30‐day and in‐hospital mortality).

### Data extraction

One researcher extracted data on study characteristics (author, year of publication, country of origin, study design, patient number), patient demographics (age, sex), tumour stage (AJCC T category and AJCC stage), method and details of anastomotic technique, and reported clinical outcomes.

### Definitions

Oesophageal cancer was defined as a malignancy of any portion of the oesophagus. Anastomotic technique was defined as any method of oesophagogastric anastomosis including HS, CS, LSSM^15^, ^16^ and TS^17^, ^18^ anastomoses. These anastomotic techniques may be employed in either the thoracic or the cervical phase of the operation. Subtle variations of the LSSM technique were described. The nomenclature includes a (modified) Collard technique^24^ and a side‐to‐side semimechanical technique, which both refer to a combination of a linear stapled and hand‐sewn technique.

### Assessment of methodological quality

Methodological quality and standard of outcome reporting within included studies were assessed by two independent researchers. Methodological quality was assessed formally using the Newcastle–Ottawa Scale (NOS)^25^, ^26^ for cohort studies and the Cochrane risk‐of‐bias tool for RCTs.

### Statistical analysis

Dichotomous outcomes were compared between anastomotic formation techniques using odds ratios (ORs), produced using random‐effects DerSimonian–Laird meta‐analytical models. Both randomized and non‐randomized studies were pooled into a network meta‐analysis comparing the above anastomotic formation techniques. Sensitivity analyses were performed for type of study (RCTs only, RCTs and prospective cohort studies (PCSs) and all RCT and cohort studies with a NOS score of 8 or above), study year (2005–2018), and level of anastomosis (cervical *versus* thoracic). For each outcome, graphical representations of treatments (nodes) and comparisons (lines) were mapped. Network maps were then analysed for closed loops to be entered into network analyses.

Networks were then examined for the presence of inconsistency, allowing for comparisons between direct and indirect treatment effects. Initially, this was assessed by checking for overall inconsistency throughout the entire network. A further check was then performed by fitting node side‐splitting models, to identify loop inconsistency, within all three‐way treatment comparison loops, as described by Dias and colleagues^27^. When *P* values were greater than 0·050, representing acceptance of the null hypothesis, consistency was assumed and networks were entered into consistency modelling. Consistency modelling utilized a restricted maximum likelihood model, generating network forest plots. Heterogeneity was examined by calculating the value of τ^2^. Hand‐sewn anastomosis was used as the common reference treatment for all comparisons. These were supplemented with interval plots of pooled effect estimates.

Anastomotic techniques were then ranked using the P‐score provided by the *netmeta* package (RStudio® 3.2.1, Boston, Massachusetts, USA; https://CRAN.R‐project.org/package=netmeta). The surface under the cumulative ranking areas for all outcomes assessed the probability of the superiority of each treatment^28^, ^29^, ^30^. The probability of ranking of a treatment (that a treatment ranks as the best treatment, second best treatment, third best treatment) for each outcome of interest was calculated. A probability of ranking below 90 per cent was not considered to be high enough to be confidently reported as the correct ranking position of a surgical technique for that outcome of interest^27^, ^31^. Statistical significance was considered when *P* < 0·050. Statistical analyses were performed using R Foundation statistical software (RStudio® 3.2.1).

## Results

**Figure 1:**
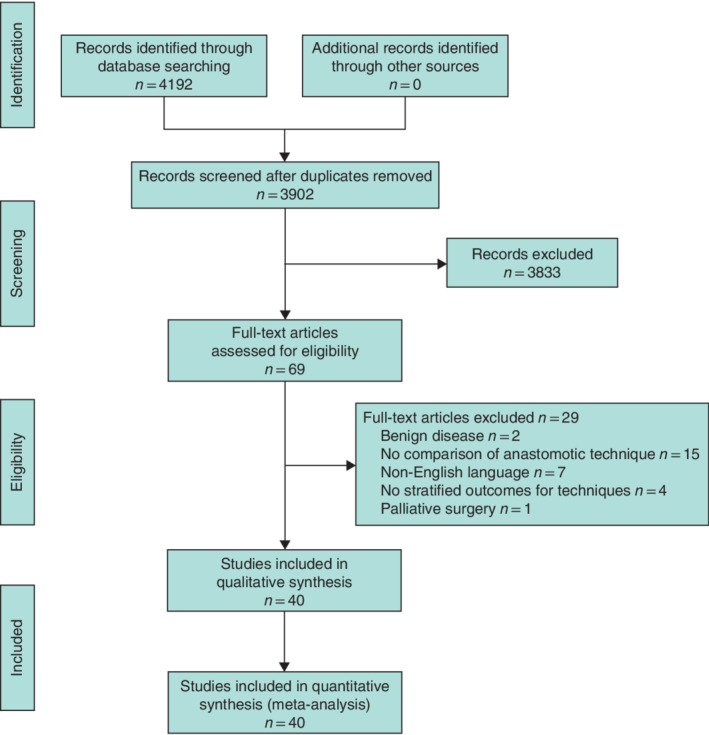
PRISMA diagram for the review

Of 4192 studies screened, 40 studies comparing different anastomotic techniques were eligible (*Fig*. *1*). Details of these studies^15^, ^16^, ^17^, ^18^, ^34^, ^35^, ^36^, ^37^, ^38^, ^39^, ^40^, ^41^, ^42^, ^43^, ^44^, ^45^, ^46^, ^47^, ^48^, ^49^, ^50^, ^51^, ^52^, ^53^, ^54^, ^55^, ^56^, ^57^, ^58^, ^59^, ^60^ are shown in *Table* *1*. The majority of included studies reported on open oesophagectomy. Three studies^57^, ^60^, ^68^ compared different types of hand‐sewn layers and were therefore excluded from meta‐analysis. The remaining 37 studies comprised 8571 patients. Fifteen RCTs, five non‐randomized prospective and 17 retrospective studies were included. Within the non‐randomized studies, the mean NOS score was 7 (range 5–9) (*Table* *2*). In RCTs, reporting of the blinding of participants and outcomes was unclear, but the risk of bias was mainly low for other domains.

**Table 1:** Characteristics of included studies

Reference	Study design	Intervention	No. of patients	% of men	Mean age (years)	Neoadjuvant therapy (%)	Tumour location	Pathology
Perrachia *et al*.^32^	PCS	CS *versus* HS	214 *versus* 28	86	60	n.r.	n.r.	Mixed
Rostas *et al*.^33^	PCS	CS *versus* HS	60 *versus* 82	82	n.r.	48	Upper 1, middle 24, lower 117	AC 110, SCC 31, other 1
McManus *et al*.^34^	RCS	CS *versus* HS	99 *versus* 122	n.r.	n.r.	n.r.	n.r.	n.r.
Lee *et al*.^35^	RCS	CS *versus* HS	141 *versus* 211	85	63	n.r.	n.r.	SCC
Honkoop *et al*.^36^	RCS	CS *versus* HS	154 *versus* 114	75	61	n.r.	Any	AC 161, SCC 92
Klink *et al*.^37^	RCS	CS *versus* HS	36 *versus* 36	89	62	51	n.r.	AC, SCC
West of Scotland and Highland Anastomosis Study Group^38^	RCT	CS *versus* HS	27 *versus* 25	n.r.	64	n.r.	n.r.	n.r.
Craig *et al*.^39^	RCT	CS *versus* HS	50 *versus* 50	61	65	n.r.	Lower 100	AC, SCC
Valverde *et al*.^40^	RCT	CS *versus* HS	78 *versus* 74	91	50	n.r.	Middle 81, lower 71	AC, SCC
Law *et al*.^41^	RCT	CS *versus* HS	61 *versus* 61	88	64	n.r.	Middle 99, lower 21, double 2	SCC
Hsu *et al*.^42^	RCT	CS *versus* HS	31 *versus* 32	90	62	52	Upper 16, middle 26, lower 21	SCC
Okuyama *et al*.^43^	RCT	CS *versus* HS	14 *versus* 18	91	64	0	Middle 23, lower 9	SCC 30, undifferentiated 2
Luechakiettisak *et al*.^44^	RCT	CS *versus* HS	58 *versus* 59	84	63	n.r.	Middle 57, lower 60	SCC
Zhang *et al*.^45^	RCT	CS *versus* HS	272 *versus* 244	58	60	0	n.r.	n.r.
Cayi *et al*.^46^	RCT	CS *versus* HS	102 *versus* 125	75	58	0	Upper/middle	n.r.
Liu *et al*.^47^	RCT	CS *versus* HS	241 *versus* 237	75	62	13	Upper 82, middle 283, lower 113	n.r.
Zhu *et al*.^48^	RCS	CS *versus* HS *versus* LHS	170 *versus* 69 *versus* 1024	80	64	NR	n.r.	Mixed
Xu *et al*.^49^	PCS	CS *versus* LSSM *versus* HS	68 *versus* 166 *versus* 59	86	60	0	Upper 5, middle 198, lower 19	AC, SCC
Blackmon *et al*.^50^	RCS	CS *versus* LSSM *versus* HS	147 *versus* 44 *versus* 23	n.r.	n.r.	n.r.	n.r.	AC, SCC
Liu *et al*.^51^	RCS	CS *versus* LSSM *versus* HS	233 *versus* 147 *versus* 78	81	63	52	Lower/GOJ	AC 345, SCC 105
Wang *et al*.^15^	RCT	CS *versus* LSSM *versus* HS	47 *versus* 45 *versus* 52	56	60	0	Middle 81, lower 18	AC 12, SCC 131, undifferentiated 1
Price *et al*.^52^	PCS	CS *versus* LSSM *versus* HS *versus* MC*	48 *versus* 260 *versus* 57	83	64	57	n.r.	AC, SCC
Li *et al*.^17^	RCS	CS *versus* TS	51 *versus* 33	81	61	10	Upper 9, middle 57, lower 18	AC, SCC
Hayata *et al*.^18^	RCT	CS *versus* TS	49 *versus* 51	77	67	57	Upper 6, middle 60, lower 34	AC, SCC
Furukawa *et al*.^53^	PCS	CS *versus* TS *versus* HS	8 *versus* 11 *versus* 12	n.r.	n.r.	n.r.	n.r.	n.r.
Wang *et al*.^54^	RCS	CS *versus* TS *versus* HS	164 *versus* 34 *versus* 192	56	NR	7	Upper 84, middle 215, lower 91	n.r.
Zieren *et al*.^55^	RCT	OLHS *versus* DLHS	107†	79	58	34	n.r.	SCC
Casson *et al*.^56^	RCS	LSSM *versus* HS	38 *versus* 53	80	63	n.r.	n.r.	AC
Behzadi *et al*.^57^	RCS	LSSM *versus* HS	75 *versus* 205	84	65	n.r.	n.r.	n.r.
Ercan *et al*.^58^	RCS	LSSM *versus* HS	85 *versus* 85	90	NR	41	Upper 5, middle 4, lower 161	AC, SCC
Kondra *et al*.^59^	RCS	LSSM *versus* HS	79 *versus* 89	85	64	24	Middle 15, lower 68, GOJ 85	AC, SCC
Harustiak *et al*.^60^	RCS	LSSM *versus* HS	281 *versus* 134	88	60	56	n.r.	AC, SCC
Mishra *et al*.^61^	RCS	LSSM *versus* HS	74 *versus* 66	56	53	0	Upper 2, middle 61, lower 62, GOJ 15	AC, SCC
Sugimura *et al*.^62^	RCS	LSSM *versus* HS	225 *versus* 173	80	n.r.	74	Upper 41, middle 229, lower 128	AC 13, SCC 381
Laterza *et al*.^63^	RCT	LSSM *versus* HS	20 *versus* 21	17	51	n.r.	Upper 10, middle 24, lower 5	AC, SCC
Walther *et al*.^64^	RCT	LSSM *versus* HS	42 *versus* 41	69	67	0	Upper 4, middle 29, lower 40	AC, SCC
Saluja *et al*.^16^	RCT	LSSM *versus* HS	87 *versus* 87	66	51	61	Middle 84, lower 80, unknown 10	AC, SCC
Singh *et al*.^65^	RCS	LSSM *versus* TS *versus* HS	16 *versus* 43 *versus* 34	n.r.	n.r.	n.r.	n.r.	Mixed
Sokouti *et al*.^66^	RCS	TLHS *versus* OLHS	228†	59	60	n.r.	Upper 13, middle 100, lower 97	Mixed
Sun *et al*.^67^	RCS	TLHS *versus* DLHS	339†	61	61	n.r.	Upper 98, middle 114, lower 127	Mixed

* Combined longitudinal and transverse anastomosis.

† Comparison of two hand‐sewn anastomosis techniques. PCS, prospective cohort study; CS, circular stapled; HS, hand‐sewn; n.r., not reported; AC, adenocarcinoma; SCC, squamous cell carcinoma; RCS, retrospective cohort study; LHS, layered hand‐sewn; LSSM, linear stapled/semimechanical; GOJ, gastro‐oesophageal junction; MC, modified Collard; TS, triangulating stapled; OLHS, one layer hand‐sewn; DLHS, double layer hand‐sewn; TLHS, triple layer hand‐sewn.

**Table 2:** Assessment of risk of bias in RCTs and cohort studies

Reference	Study design	Adequate sequence generation	Allocation concealment	Blinding of participants	Blinding of outcomes	Incomplete outcome data	Selective outcome reporting	Free from other bias	NOS score
**RCTs**									
West of Scotland and Highland Anastomosis Study Group^38^	RCT	Low	Low	Unclear	Unclear	Unclear	Low	Low	–
Craig *et al*.^39^	RCT	High	High	Unclear	Unclear	Unclear	Low	Low	–
Valverde *et al*.^40^	RCT	Low	Unclear	Unclear	Unclear	Unclear	Low	Low	–
Law *et al*.^41^	RCT	Low	Low	Unclear	Unclear	Low	Low	Low	–
Hsu *et al*.^42^	RCT	High	High	Unclear	Unclear	Low	Low	Low	–
Okuyama *et al*.^43^	RCT	Low	Unclear	Unclear	Unclear	Unclear	Low	High	–
Luechakiettisak *et al*.^44^	RCT	High	High	Unclear	Unclear	Low	Low	Low	–
Zhang *et al*.^45^	RCT	High	High	Unclear	Unclear	Unclear	Low	Low	–
Cayi *et al*.^46^	RCT	Uncertain	Unclear	Unclear	Unclear	Unclear	Low	High	–
Liu *et al*.^47^	RCT	Low	Low	Unclear	Low	Unclear	Low	Low	–
Wang *et al*.^15^	RCT	High	High	Unclear	Unclear	Low	Low	Low	–
Hayata *et al*.^18^	RCT	Uncertain	Low	Unclear	Unclear	Low	Low	High	–
Zieren *et al*.^55^	RCT	Low	Low	Unclear	Unclear	Unclear	Unclear	Low	–
Laterza *et al*.^63^	RCT	Low	Unclear	Unclear	Unclear	Low	Low	Low	–
Walther *et al*.^64^	RCT	Low	Unclear	Unclear	Unclear	Low	Low	High	–
Saluja *et al*.^16^	RCT	Low	Unclear	Unclear	Unclear	Low	Low	Low	–
**Cohort studies**									
Perrachia *et al*.^32^	PCS	–	–	–	–	–	–	–	7
Rostas *et al*.^33^	PCS	–	–	–	–	–	–	–	7
McManus *et al*.^34^	RCS	–	–	–	–	–	–	–	6
Lee *et al*.^35^	RCS	–	–	–	–	–	–	–	6
Honkoop *et al*.^36^	RCS	–	–	–	–	–	–	–	9
Klink *et al*.^37^	RCS	–	–	–	–	–	–	–	7
Zhu *et al*.^48^	RCS	–	–	–	–	–	–	–	6
Xu *et al*.^49^	PCS	–	–	–	–	–	–	–	9
Blackmon *et al*.^50^	RCS	–	–	–	–	–	–	–	7
Liu *et al*.^51^	RCS	–	–	–	–	–	–	–	7
Price *et al*.^52^	PCS	–	–	–	–	–	–	–	8
Li *et al*.^17^	RCS	–	–	–	–	–	–	–	6
Furukawa *et al*.^53^	PCS	–	–	–	–	–	–	–	8
Wang *et al*.^54^	RCS	–	–	–	–	–	–	–	7
Casson *et al*.^56^	RCS	–	–	–	–	–	–	–	7
Behzadi *et al*.^57^	RCS	–	–	–	–	–	–	–	6
Ercan *et al*.^58^	RCS	–	–	–	–	–	–	–	n.a.
Kondra *et al*.^59^	RCS	–	–	–	–	–	–	–	8
Harustiak *et al*.^60^	RCS	–	–	–	–	–	–	–	9
Mishra *et al*.^61^	RCS	–	–	–	–	–	–	–	8
Sugimura *et al*.^62^	RCS	–	–	–	–	–	–	–	8
Singh *et al*.^65^	RCS	–	–	–	–	–	–	–	5
Sokouti *et al*.^66^	RCS	–	–	–	–	–	–	–	7
Sun *et al*.^67^	RCS	–	–	–	–	–	–	–	7

Level of bias was determined as: low, indicating a low risk of bias; unclear, indicating an uncertain risk of bias, and high, indicating a high risk of bias. NOS, Newcastle–Ottawa Scale; PCS, prospective cohort study; RCS, retrospective cohort study; n.a., not applicable.

The anastomotic techniques analysed most commonly were HS (35 studies) and CS (26), with these two techniques being directly compared in 24 studies. LSSM was analysed in 16 studies, of which ten included comparisons with HS, and a further five studies reported comparisons with both HS and CS. In addition, five studies analysed TS anastomoses. One study^52^ described a linear stapled technique without the use of a hand‐sewn component. A summary of technical details of anastomosis is presented in *Table S2* (supporting information).

### Network meta‐analysis

**Figure 2:**
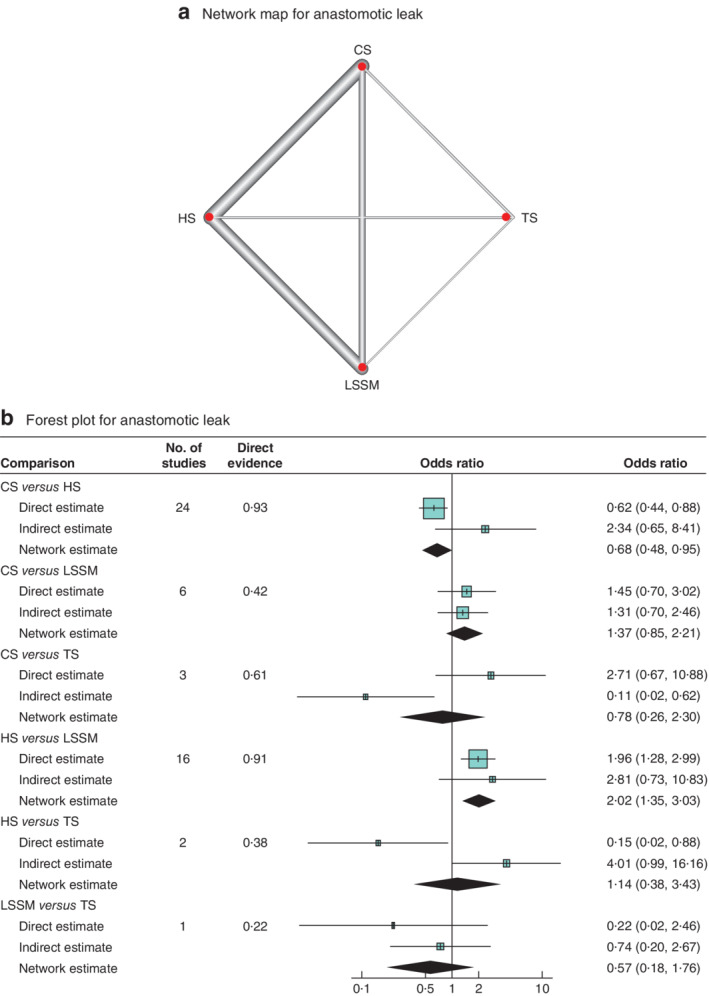
Network map and forest plot for anastomotic leak **a** Network map. **b** Forest plot. A DerSimonian–Laird random‐effects model was used for meta‐analysis. Odds ratios are shown with 95 per cent confidence intervals. CS, circular stapled; HS, hand‐sewn; TS, triangulating stapled; LSSM, linear stapled/semimechanical.

**Figure 3:**
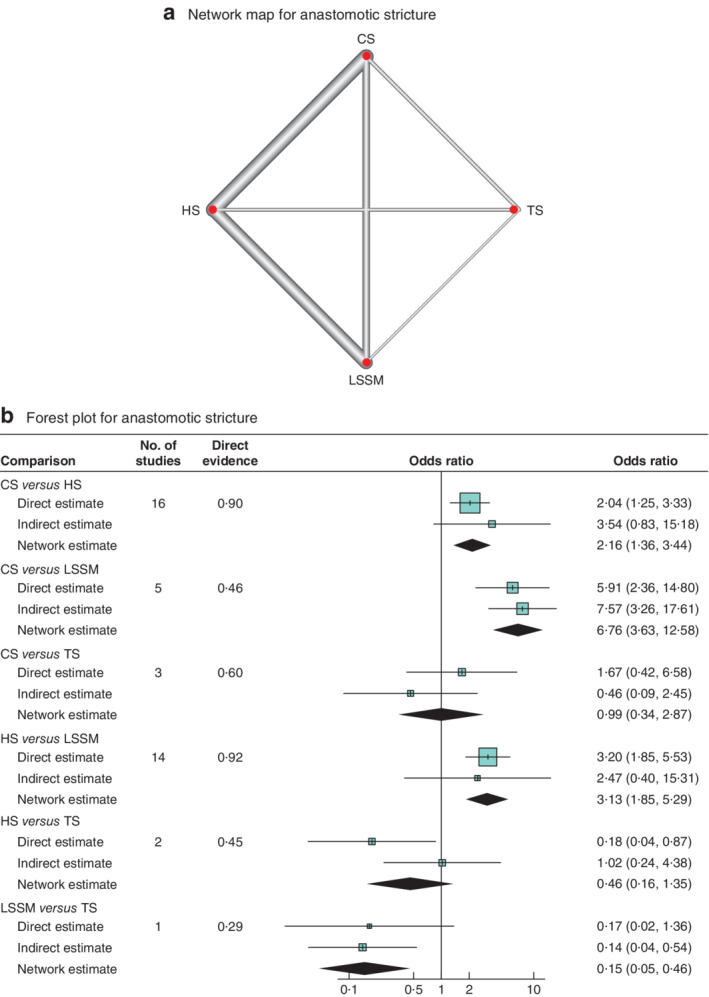
Network map and forest plot for anastomotic stricture **a** Network map. **b** Forest plot. A DerSimonian–Laird random‐effects model was used for meta‐analysis. Odds ratios are shown with 95 per cent confidence intervals. CS, circular stapled; HS, hand‐sewn; TS, triangulating stapled; LSSM, linear stapled/semimechanical.

Network meta‐analyses for the two primary outcomes, anastomotic leak and stricture, were conducted comparing all anastomotic techniques described in two or more studies including HS, TS, LSSM and CS. Initially, visual representations of the network of studies used for each outcome were generated (*Figs* *2a* and *3a*). For anastomotic leak, 36 studies were included, consisting of 2623 patients with a CS anastomosis, 1876 with a LSSM anastomosis, 3922 receiving a HS anastomosis, and 197 who had a TS anastomosis. For anastomotic stricture formation, 27 studies were included, comprising 1775 patients with a CS anastomosis, 1602 with a LSSM anastomosis, 3224 with a HS anastomosis, and 197 receiving a TS anastomosis.

### Anastomotic leak

After overall inconsistency testing (*P* = 0·958) and fitting of node side‐splitting models (*P* values: 0·565, 0·972, 0·916, 0·715, 0·743, 0·617), overall and local consistency was assumed. Upon generation of network forest and interval plots (*Fig*. *2b* and *Table* *3*), CS (OR 0·68; *P* = 0·027) and LSSM (OR 0·50; *P* = 0·001) anastomoses were associated with lower anastomotic leak rates than HS anastomosis. Anastomotic leak rates were no different for CS and LSSM anastomoses (OR 1·37; *P* = 0·198). There were no significant differences in leak rates between TS and HS. Overall, LSSM was ranked the best technique regarding anastomotic leakage with high probability, followed by CS and TS (*Table S3*, supporting information).

**Table 3:** Results of network meta‐analysis of all techniques for anastomotic leak and benign anastomotic stricture formation, for overall studies and subgroup analyses

	Anastomotic leak			Anastomotic stricture		
	No. of studies	Odds ratio	*P*	No. of studies	Odds ratio	*P*
**All studies**						
CS *versus* HS	24	0·68 (0·48, 0·95)	0·027	16	2·16 (1·36, 3·44)	0·001
LSSM *versus* CS	6	0·73 (0·45, 1·18)	0·198	5	0·15 (0·08, 0·28)	< 0·001
CS *versus* TS	3	0·78 (0·26, 2·30)	0·668	3	0·99 (0·34, 2·87)	0·987
LSSM *versus* HS	16	0·50 (0·33, 0·74)	0·001	14	0·32 (0·19, 0·54)	< 0·001
HS *versus* TS	2	1·14 (0·38, 3·43)	0·827	2	0·46 (0·16, 1·35)	0·154
LSSM *versus* TS	1	0·57 (0·18, 1·76)	0·339	1	0·15 (0·05, 0·46)	0·001
**Level of anastomosis**						
Cervical						
CS *versus* HS	6	1·04 (0·49, 2·20)	0·925	4	2·86 (1·11, 7·37)	0·029
LSSM *versus* CS	1	0·50 (0·19, 1·30)	0·153	1	0·10 (0·03, 0·32)	< 0·001
CS *versus* TS	2	1·18 (0·29, 4·83)	0·829	2	1·19 (0·31, 4·60)	0·812
LSSM *versus* HS	8	0·52 (0·26, 1·02)	0·058	8	0·30 (0·13, 0·65)	0·002
HS *versus* TS	1	1·13 (0·27, 4·78)	0·877	1	0·42 (0·10, 1·66)	0·228
LSSM *versus* TS	1	0·59 (0·13, 2·64)	0·502	1	0·12 (0·03, 0·53)	0·004
Thoracic						
CS *versus* HS	7	0·56 (0·38, 0·83)	0·004	6	1·95 (0·66, 5·78)	0·23
LSSM *versus* CS	3	0·75 (0·42, 1·37)	0·353	3	0·14 (0·03, 0·63)	0·01
CS *versus* TS	1	0·44 (0·07, 2·73)	0·386	1	0·97 (0·06, 15·85)	0·985
LSSM *versus* HS	4	0·42 (0·26, 0·69)	0·001	4	0·28 (0·07, 1·12)	0·072
HS *versus* TS	1	0·78 (0·12, 4·93)	0·805	1	0·50 (0·03, 8·42)	0·643
LSSM *versus* TS	0	0·33 (0·05, 2·21)	0·254	0	0·14 (0·01, 2·99)	0·177
**Study type**						
RCT only						
CS *versus* HS	11	0·72 (0·42, 1·24)	0·237	7	1·92 (0·99, 3·72)	0·053
LSSM *versus* CS	1	1·52 (0·49, 4·76)	0·484	1	0·20 (0·04, 1·02)	0·053
CS *versus* TS	1	5·68 (0·52, 61·94]	0·155	1	0·91 (0·16, 5·04)	0·922
LSSM *versus* HS	4	1·09 (0·40, 2·94)	0·880	3	0·39 (0·09, 1·75)	0·224
HS *versus* TS	0	7·92 (0·68, 91·83)	0·098	0	0·47 (0·08, 2·97)	0·421
LSSM *versus* TS	0	8·59 (0·61, 120·59)	0·111	0	0·19 (0·02, 1·95)	0·156
RCT + cohort studies (NOS score ≥ 8)						
CS *versus* HS	15	0·68 (0·46, 1·01)	0·054	11	1·91 (1·18, 3·10)	0·009
LSSM *versus* CS	3	0·81 (0·48, 1·39)	0·453	3	0·14 (0·07, 0·28)	< 0·001
CS *versus* TS	2	1·31 (0·30, 5·70)	0·732	2	0·95 (0·26, 3·42)	0·943
LSSM *versus* HS	10	0·56 (0·38, 0·82)	0·003	9	0·28 (0·16, 0·48)	< 0·001
HS *versus* TS	1	1·92 (0·43, 8·60)	0·400	1	0·50 (0·13, 1·91)	0·317
LSSM *versus* TS	0	1·07 (0·23, 4·99)	0·937	0	0·14 (0·03, 0·57)	0·009
**Study year (2005–2018)**						
CS *versus* HS	15	0·70 (0·45, 1·10)	0·118	12	1·77 (1·01, 3·11)	0·046
LSSM *versus* CS	6	0·70 (0·41, 1·20)	0·197	5	0·15 (0·07, 0·30)	< 0·001
CS *versus* TS	3	1·86 (0·49, 7·02)	0·367	3	1·41 (0·36, 5·46)	0·633
LSSM *versus* HS	12	0·50 (0·32, 0·76)	0·002	11	0·26 (0·14, 0·47)	< 0·001
HS *versus* TS	1	2·65 (0·67, 10·47)	0·165	1	0·80 (0·19, 3·35)	0·773
LSSM *versus* TS	0	1·31 (0·32, 5·38)	0·721	0	0·21 (0·05, 0·93)	0·036

Values in parentheses are percentages. CS, circular stapled; HS, hand‐sewn; LSSM, linear stapled/semimechanical; TS, triangulating stapled; NOS, Newcastle–Ottawa Scale.

### Sensitivity analysis of anastomotic leak

For cervical anastomosis, no technique was superior with regard to anastomotic leakage (*Table* *3*). For thoracic anastomosis, LSSM (OR 0·42) and CS (OR 0·56) anastomoses were superior to HS with regard to anastomotic leakage. There were no differences in anastomotic leaks between CS and LSSM (OR 1·33). In the analyses split by study type, only LSSM anastomoses (OR 0·56) had a lower anastomotic leak rate than HS in the ‘RCT and cohort studies with a NOS score of 8 or above’ subgroup only. In the subgroup analysis of RCTs there were no statistically significant differences. For studies published in 2005–2018, only LSSM (OR 0·50) was superior to HS anastomosis.

### Anastomotic stricture

After overall inconsistency testing (*P* = 0·425) and fitting of node side‐splitting models (*P* values: 0·995, 0·124, 0·516, 0·413, 0·782), overall and local consistency was assumed. Upon generation of network forest and interval plots (*Fig*. *3b* and *Table* *3*), LSSM anastomosis was found to be superior to CS (OR 0·15; *P* < 0·001), HS (OR 0·32; *P* < 0·001) and TS (OR 0·15; *P* = 0·001) anastomoses respectively. CS was inferior to HS (OR 2·16; *P* = 0·001). LSSM was ranked the best technique with high probability followed by HS, TS and CS anastomoses respectively.

### Sensitivity analysis in anastomotic stricture

For cervical anastomosis, LSSM had lower rates of anastomotic stricture than CS (OR 0·10; *P* < 0·001), HS (OR 0·30; *P* = 0·002) and TS (OR 0·12; *P* = 0·004) anastomoses (*Table* *3*). CS had higher rates of anastomotic stricture than HS (OR 2·86; *P* = 0·029). For thoracic anastomosis, LSSM had lower rates of anastomotic stricture than CS anastomosis (OR 0·14; *P* = 0·010). There were no significant differences in anastomotic stricture between CS and HS.

By study type, no significant differences were noted in the RCT‐only sensitivity analysis. LSSM was superior to TS, CS and HS for anastomotic strictures in the ‘RCT and cohort studies with a NOS score of 8 or above’ subgroup only. CS had significantly higher rates of stricture than LSSM and HS. For studies published in 2005–2018, LSSM was superior to CS (OR 0·15; *P* < 0·001), HS (OR 0·26; *P* < 0·001) and TS (OR 0·21; *P* = 0·036) anastomoses (*Table* *3*).

### Intraoperative outcomes

Duration of surgery was reported in 16 studies. There were no differences in operating times between techniques (*Table S4*, supporting information). LSSM was ranked first for the entire cohort and for cervical anastomosis only. Blood loss was reported in 11 studies. LSSM had significantly lower blood loss than HS (mean difference 24 ml; *P* = 0·024). LSSM was ranked first for the entire cohort and for cervical anastomosis only. There were insufficient studies in the thoracic anastomosis subgroup only for analysis.

### Other postoperative complications

Cardiac complications rates were reported in ten studies (CS *versus* HS, 7 studies; LSSM *versus* HS, 2; CS *versus* TS, 1) (*Table S4*, supporting information). There were no significant differences in cardiac complications between the different techniques. LSSM was ranked first for the overall and cervical anastomosis only subgroup. There were not enough studies in the thoracic anastomosis only subgroup for analysis.

Pulmonary complications were reported in 12 studies (CS *versus* HS, 8 studies; LSSM *versus* HS, 2; CS *versus* TS, 2). There were no significant differences in pulmonary complications between the different techniques. TS was ranked first in the overall group. LSSM was ranked first in the cervical anastomosis only subgroup. There were not enough studies in the thoracic anastomosis only subgroup for analysis.

Thirty‐day mortality was reported in 11 studies (CS *versus* HS, 7 studies; LSSM *versus* HS, 3; CS *versus* TS, 1). LSSM was associated with lower rates of 30‐day mortality than HS (OR 0·33; *P* = 0·016) and CS (OR 0·18; *P* = 0·002) anastomoses. CS was not associated with higher mortality rates than HS anastomosis. LSSM was ranked the best technique with high probability, followed by TS. For cervical anastomosis, LSSM was ranked first. There were not enough studies in the thoracic anastomosis only subgroup for analysis.

In‐hospital mortality was reported in several pairwise comparisons: CS *versus* HS, 16 studies; LSSM *versus* HS, five; CS *versus* TS, three; LSSM *versus* HS, four studies. LSSM was associated with lower rates of in‐hospital mortality than HS (OR 0·32; *P* < 0·001), CS (OR 0·15; *P* < 0·001) and TS (OR 0·15; *P* = 0·001) anastomoses. CS was associated with higher in‐hospital mortality rates than HS anastomosis. LSSM was ranked the best technique with high probability, followed by HS. For cervical anastomosis, HS was ranked first. There were not enough studies in the thoracic anastomosis only subgroup for analysis.

## Discussion

This study demonstrates that stapled anastomoses, specifically using an LSSM technique, are associated with lower anastomotic leak rates than HS anastomoses following oesophagectomy. The LSSM technique was associated with a lower rate of anastomotic stricture than CS, TS and HS anastomoses. This effect was consistent across the majority of subgroups in sensitivity analyses. LSSM anastomoses were associated with lower rates of 30‐day mortality. Overall, the results indicate superiority of the LSSM technique for oesophagogastric anastomosis following oesophagectomy.

Previous systematic reviews and meta‐analyses^10^, ^11^, ^12^, ^13^, ^14^ have examined the impact of stapled *versus* HS anastomoses following oesophagectomy (*Table S5*, supporting information). These, however, did not distinguish between CS and LSSM stapling techniques. Honda and colleagues^10^ did not include LSSM anastomoses, looking only at the differences between HS and CS anastomoses. They reported no differences in anastomotic leak rates but an increased risk of anastomotic stricture with CS. Wang and co‐workers^13^ only compared HS with CS anastomoses. All anastomoses were performed in the neck, and no significant differences were demonstrated with regard to anastomotic leak, stricture or mortality. Markar *et al*.^14^ compared HS with stapled oesophagogastric anastomoses, but did not separate the types of stapled anastomosis further. They did not observe significant differences in anastomotic leakage or 30‐day mortality. Anastomotic stricture occurred more frequently with stapled than with HS anastomoses. Another systematic review^11^ examined eight RCTs, all comparing HS with CS anastomoses. No meta‐analysis was performed, and the authors concluded that there was insufficient evidence to recommend either technique. Liu and colleagues^12^ grouped all stapled anastomoses together and compared them with HS anastomoses. Although a number of subgroup analyses were performed, the overall results demonstrated no significant differences in anastomotic leak rates or 30‐day mortality between HS and stapled anastomoses.

Although the data from the present study are interesting, it is not known precisely why LSSM anastomoses may have a reduced anastomotic leak rate. Theories include: the wider anastomosis, hence also reducing the risk of anastomotic stricture; anastomosis performed near the greater curve arcade on the best perfused part of the stomach allows for improved healing; and the side‐to‐side orientation reduces traction‐related tension^24,58^.

The present study has some limitations. The studies included in the review span a large time scale of over 28 years, and used slightly different definitions of anastomotic leakage. Future trials and studies in this area should adhere to the Esophagectomy Complications Consensus Group definitions of anastomotic leakage^68^, which classify leaks into three types in relation to severity and treatment needs. There is only limited published evidence on the use of the LSSM anastomoses, and this tends to be from more recently conducted studies. The included studies are heterogeneous in that they included different levels of anastomosis, suture material used, stapling device types and sizes, and approaches employed for oesophagectomy (open *versus* minimal access).

In the absence of large, high‐quality, randomized trial data, this network meta‐analysis provides the most up‐to‐date evidence base for comparing HS *versus* LSSM and CS techniques. Although triangular stapling showed promising results in the overall network, the encouraging results were not consistent in the sensitivity analysis. There is limited literature on the TS method, with only two papers describing outcomes for this technique; therefore it is difficult to give recommendations. The TS technique may be better viewed as a variation of the LSSM technique. The current multicentre international Oesophago‐Gastric Anastomosis Audit^69^ (https://www.ogaa.org.uk) is collecting data on outcomes after oesophagectomy, and includes intraoperative details regarding anastomotic techniques. Data from this large database will further inform surgical teams about the benefits of different anastomotic techniques.

## Supporting information


**Table S1** Search terms
**Table S2** Technical details of anastomoses reported
**Table S3** Summary of studies reporting outcomes included in meta‐analysis
**Table S4** Summary of other intraoperative and postoperative outcomes included in network meta‐analysis
**Table S5** Summary of outcomes in previously published meta‐analyses
