# Supplementary material for: Anastomotic techniques for oesophagectomy for malignancy: systematic review and network meta‐analysis
Source: BJS Open. 2020 May 23;4(4):563–76. doi: 10.1002/bjs5.50298 (PMC7397345; doi:10.1002/bjs5.50298)
Supplement: Supplementary file 1 — Table S1 Search terms Table S2 Technical details of anastomoses reported Table S3 Summary of studies reporting outcomes included in meta‐analysis Table S4 Summary of other intraoperative and postoperative outcomes included in network meta‐analysis Table S5 Summary of outcomes in previously published meta‐analyses [file BJS5-4-563-s001.docx]

**BJS5_50298**

**Anastomotic techniques for oesophagectomy for malignancy: systematic review and network meta-analysis**

**S. K. Kamarajah, J. R. Bundred, P. Singh, S. Pasquali and E. A. Griffiths**

**Table S1** Search terms

| 1 | exp Esophagectomy/ | 28813 |
| --- | --- | --- |
| 2 | oesophagectomy.ti. | 2113 |
| 3 | esophagectomy.ti. | 10457 |
| 4 | exp general surgery/ or exp surgical oncology/ | 52424 |
| 5 | surgery.ti,ab. | 2921853 |
| 6 | 1 or 2 or 3 or 4 or 5 | 2971887 |
| 7 | exp Esophagogastric Junction/ | 11272 |
| 8 | exp NEOPLASMS/ | 7329709 |
| 9 | exp Esophageal Neoplasms/ | 123171 |
| 10 | esophageal cancer.ti,ab. | 46532 |
| 11 | oesophageal cancer.ti,ab. | 8541 |
| 12 | 7 and 8 | 4513 |
| 13 | 9 or 10 or 11 or 12 | 138423 |
| 14 | exp Suture Techniques/ | 47533 |
| 15 | exp Surgical Staplers/ or exp Surgical Stapling/ | 19644 |
| 16 | circular stapled.ti,ab. | 563 |
| 17 | hand sewn.ti,ab. | 2917 |
| 18 | stapler.ti,ab. | 11010 |
| 19 | exp Anastomosis, Surgical/ | 262058 |
| 20 | anastomosis.ti,ab. | 141213 |
| 21 | anastomoses.ti,ab. | 41093 |
| 22 | 14 or 15 or 16 or 17 or 18 or 19 or 20 or 21 | 411051 |
| 23 | 6 and 13 and 22 | 4192 |

**Table S2** Technical details of anastomoses reported

|  |  | **Circular Stapler** | | | | **Linear Stapler / Semi-mechanical** | | | | **Hand-Sewn** | | | | **Triangulating Stapler** | | | |
| --- | --- | --- | --- | --- | --- | --- | --- | --- | --- | --- | --- | --- | --- | --- | --- | --- | --- |
| **Study Name** | **Intervention** | **Brand** | **Staple Size, mm** | **Configuration** | **Site** | **Brand** | **Staple Size, mm** | **Configuration** | **Site** | **Suture Material** | **Layer** | **Configuration** | **Site** | **Brand** | **Staple Size, mm** | **Configuration** | **Site** |
| Perrachia 1988^32^ | CS vs HS | Mixed | NR | NR | NR | - | - | - | - | Mixed | NR | NR | NR | - | - | - | - |
| Rostas 2018^33^ | CS vs HS | EEA | 25, 29 | End-to-End | Thoracic | - | - | - | - | Absorbable | Double | Side-to-Side | Thoracic |  | - | - | - |
| McManus 1990^34^ | CS vs HS | Mixed | NR | NR | NR | - | - | - | - | Mixed | NR | NR | NR | - | - | - | - |
| Lee 1992^35^ | CS vs HS | ILS/EEA | NR | End-to-end / End-to-side | Thoracic | - | - | - | - | Absorbable | Mixed | End-to-end | Thoracic | - | - | - | - |
| Honkoop 1996^36^ | CS vs HS | EEA | 21,25 | NR | Cervical | - | - | - | - | Absorbable | Single | NR | Cervical | - | - | - | - |
| Klink 2012^37^ | CS vs HS | NR | NR | NR | NR | - | - | - | - | NR | NR | End-to-end | Cervical | - | - | - | - |
| WSHASG 1991^38^ | CS vs HS | EEA | NS | NR | NR | - | - | - | - | Mixed | Mixed | NR | NR | - | - | - | - |
| Craig 1996^39^ | CS vs HS | ILS | NR | End-to-end | Thoracic | - | - | - | - | Mixed | Mixed | NS | NS | - | - | - | - |
| Valverde 1996^40^ | CS vs HS | Mixed | NR | Mixed | Mixed | - | - | - | - | Mixed | Mixed | Mixed | Mixed | - | - | - | - |
| Law 1997^41^ | CS vs HS | EEA, ILS | 25, 28, 29, 31, 33 | End-to-side | Cervical | - | - | - | - | Absorbable | Single | End-to-side | Cervical | - | - | - | - |
| Hsu 2004^42^ | CS vs HS | ILS | 21 | End-to-side | Cervical | - | - | - | - | Absorbable, No-bsorbable | Double | End-to-side | Cervical | - | - | - | - |
| Okuyama 2007^43^ | CS vs HS | PCEEA | 25 | End-to-side | Thoracic | - | - | - | - | Absorbable, No-bsorbable | Double | End-to-side | Cervical | - | - | - | - |
| Luechakiettisak 2008^44^ | CS vs HS | ILS | 25, 31 | NR | Cervical | - | - | - | - | Absorbable | Single | NR | Cervical | - | - | - | - |
| Zhang 2009^45^ | CS vs HS | ILS | 25 | NR | Mixed | - | - | - | - | Absorbable, No-bsorbable | Double | NR | Mixed | - | - | - | - |
| Cayi 2012^46^ | CS vs HS | NR | NR | NR | Cervical | - | - | - | - | NR | NR | NR | Cervical | - | - | - | - |
| Liu 2015^47^ | CS vs HS | ILS | 25, 29, 33 | End-to-end | Mixed | - | - | - | - | Absorbable | Single | End-to-end | Mixed | - | - | - | - |
| Zhu 2008^48^ | CS vs HS vs LHS | EEA | 25 | NR | Thoracic | - | - | - | - | Absorbable | Double | NR | Thoracic | Three leaf clipper | 4/0 vicryl | - | - |
| Xu 2011^49^ | CS vs LSSM vs HS | EEA | 25 | NR | Thoracic | TLC | TLC75 | Side-to-side | Thoracic | Absorbable | Single | NR | Thoracic | - | - | - | - |
| Blackmon 2007^50^ | CS vs LSSM vs HS | CDH | 25,29,33 | End-to-side | Thoracic | Endo-GIA | NR | NR | NR | Absorbable | double | End-to-side | Thoracic | - | - | - | - |
| Liu 2018^51^ | CS vs LSSM vs HS | NR | NR | NR | NR | NR | NR | NR | NR | NR | NR | NR | NR | - | - | - | - |
| Wang 2013^15^ | CS vs LSSM vs HS | CDH, SDH | NR | Side-to-side | Thoracic | TLC75 | NR | Side-to-side | Thoracic | NR | Single | Side-to-side | Thoracic | - | - | - | - |
| Price 2013^52^ | CS vs LSSM vs HS vs MC* | NR | NR | NR | NR | NR | NR | NR | NR | NR | NR | NR | NR | NR | NR | NR | NR |
| Li 2014^17^ | CS vs TS | NR | NR | End-to-side | Cervical | - | - | - | - | - | - | - | - | NR | NR | End-to-end | Thoracic |
| Hayata 2017^18^ | CS vs TS | CDH25 | 25 | End-to-side | Cervical | - | - | - | - | - | - | - | - | NR | NR | End-to-end | Cervical |
| Furukawa 2005^53^ | CS vs TS vs HS | EEA | 25 | NR | Thoracic | - | - | - | - | Absorbable | NR | NR | Thoracic | - | - | - | - |
| Wang 2018^54^ | CS vs TS vs HS | NR | NR | NR | Cervical | ETS60 | 3.5 | Side-to-side | Cervical | Absorbable | Double | Side-to-Side | Cervical |  | - | - | - |
| Zieren 1993^55^ | SLHS vs DLHS | - | - | - | - | - | - | - | - | Absorbable | Single | End-to-end | Cervical | - | - | End-to-end | Cervical |
| Casson 2002^56^ | LSSM vs HS | - | - | - | - | ETS | 45 | Side-to-side | Cervical | Absorbable | mixed | NR | Cervical | - | - | - | - |
| Behzadi 2005^57^ | LSSM vs HS | NR | NR | NR | NR | NR | NR | NR | NR | NR | NR | NR | NR | NR | NR | NR | NR |
| Ercan 2005^58^ | LSSM vs HS | - | - | - | - | EndoGIA | NR | Side-to-side | Cervical | Absorbable | mixed | End to Side | Cervical | - | - | - | - |
| Kondra 2008^59^ | LSSM vs HS | - | - | - | - | EndoGIA | NR | Side-to-side | Cervical | Absorbable | double | NR | Cervical | - | - | - | - |
| Harustiak 2015^60^ | LSSM vs HS | - | - | - | - | EndoGIA | NR | Side-to-side | Thoracic | Absorbable | Single/double | End-to-end | Thoracic | - | - | - | - |
| Mishra 2016^61^ | LSSM vs HS | - | - | - | - | EndoGIA | NR | Side-to-side | Cervical | Absorbable | Single | End-to-end | Cervical | - | - | - | - |
| Sugimiura 2019^62^ | LSSM vs HS | - | - | - | - | NR | NR | Side-to-side | Cervical | Absorbable | Double | NR | Cervical |  | - | - | - |
| Laterza 1999^63^ | LSSM vs HS | - | - | - | - | NR | 21, 25 | End-to-side | Cervical | Absorbable | Double | End-to-end | Cervical | - | - | - | - |
| Walther 2003^64^ | LSSM vs HS | - | - | - | - | PCEEA | 25, 28, 31 | End-to-side | Thoracic | Absorbable | Single | End-to-end | Cervical | - | - | - | - |
| Saluja 2012^16^ | LSSM vs HS | - | - | - | - | Endopath EZ45 | NR | Side-to-side | Cervical | Absorbable | Double | End-to-side | Cervical | - | - | - | - |
| Singh 2001^65^ | LSSM vs TS vs HS | - | - | - |  | EndoGIA | NR | Side-to-side | Cervical | Absorbable | Single | End-to-side | Cervical | EndoGIA | NR | Side-to-side | Cervical |
| Sokouti 2013^66^ | TLHS vs OLHS | - | - | - | - | - | - | - | - | Absorbable | Single | End-to-end | Cervical | - | - | - | - |
| Sun 2016^67^ | TLHS vs DLHS | - | - | - | - | - | - | - | - | Absorbable | Double vs Triple | End-to-end | Cervical | - | - | - | - |

**Table S3** Summary of studies reporting outcomes included in meta-analysis

| Study Name | Intervention | Operating Time | Blood Loss | Anastomotic Leaks | Anastomotic Strictures | Cardiac Complications | Pulmonary Complications | 30-day Mortality | In-hospital Mortality |
| --- | --- | --- | --- | --- | --- | --- | --- | --- | --- |
| Perrachia 1988 | CS vs HS | No | No | Yes | No | No | No | No | Yes |
| Rostas 2018 | CS vs HS | No | Yes | Yes | Yes | No | No | No | Yes |
| McManus 1990 | CS vs HS | No | No | Yes | Yes | No | No | No | No |
| Lee 1992 | CS vs HS | No | No | Yes | No | No | No | No | No |
| Honkoop 1996 | CS vs HS | No | No | Yes | Yes | No | No | No | No |
| Klink 2012 | CS vs HS | Yes | No | Yes | No | No | No | No | Yes |
| WSHASG 1991 | CS vs HS | Yes | No | Yes | No | No | No | Yes | Yes |
| Craig 1996 | CS vs HS | Yes | Yes | Yes | Yes | Yes | Yes | Yes | Yes |
| Valverde 1996 | CS vs HS | Yes | No | Yes | No | Yes | Yes | Yes | No |
| Law 1997 | CS vs HS | Yes | Yes | Yes | No | Yes | Yes | Yes | Yes |
| Hsu 2004 | CS vs HS | Yes | No | Yes | Yes | Yes | Yes | Yes | Yes |
| Okuyama 2007 | CS vs HS | Yes | Yes | Yes | Yes | No | Yes | No | Yes |
| Luechakiettisak 2008 | CS vs HS | Yes | Yes | Yes | No | Yes | Yes | Yes | No |
| Zhang 2009 | CS vs HS | Yes | No | Yes | Yes | No | No | Yes | No |
| Cayi 2012 | CS vs HS | No | No | Yes | Yes | No | No | Yes | Yes |
| Liu 2015 | CS vs HS | Yes | No | Yes | Yes | Yes | Yes | No | No |
| Zhu 2008 | CS vs HS vs LHS | Yes | No | Yes | Yes | Yes | Yes | No | Yes |
| Xu 2011 | CS vs LSSM vs HS | No | No | Yes | Yes | No | No | No | Yes |
| Blackmon 2007 | CS vs LSSM vs HS | No | No | Yes | Yes | No | No | No | No |
| Liu 2018 | CS vs LSSM vs HS | No | No | Yes | No | No | No | No | No |
| Wang 2013 | CS vs LSSM vs HS | No | No | Yes | Yes | No | No | Yes | Yes |
| Price 2013 | CS vs LSSM vs HS vs MC | No | No | Yes | Yes | No | No | No | No |
| Li 2014 | CS vs TS | No | No | Yes | Yes | NO | Yes | Yes | No |
| Hayata 2017 | CS vs TS | Yes | Yes | Yes | Yes | Yes | Yes | Yes | Yes |
| Furukawa 2005 | CS vs TS vs HS | No | No | Yes | Yes | No | No | No | No |
| Wang 2018 | LSSM vs CS vs HS | No | No | Yes | Yes | No | No | No | Yes |
| Casson 2002 | LSSM vs HS | No | No | Yes | Yes | No | No | No | No |
| Behzadi 2005 | LSSM vs HS | No | No | Yes | Yes | No | No | No | No |
| Ercan 2005 | LSSM vs HS | No | No | No | No | No | No | No | No |
| Kondra 2008 | LSSM vs HS | No | No | Yes | Yes | No | No | Yes | No |
| Harustiak 2015 | LSSM vs HS | No | No | Yes | Yes | No | No | Yes | No |
| Mishra 2016 | LSSM vs HS | Yes | Yes | Yes | Yes | No | No | No | Yes |
| Sugimura 2019 | LSSM vs HS | Yes | Yes | Yes | Yes | Yes | Yes | Yes | No |
| Laterza 1999 | LSSM vs HS | No | No | Yes | Yes | No | No | Yes | Yes |
| Walther 2003 | LSSM vs HS | Yes | Yes | Yes | No | Yes | Yes | Yes | Yes |
| Saluja 2012 | LSSM vs HS | Yes | Yes | Yes | Yes | No | No | No | Yes |
| Singh 2001 | LSSM vs TS vs HS | No | No | Yes | Yes | No | No | No | No |
| Zieren 1993 | SLHS vs DLHS | No | No | Yes | Yes | No | No | No | Yes |
| Sokouti 2013 | THS vs OHS | No | No | Yes | No | No | No | No | Yes |
| Sun 2016 | TLHS vs DLHS | No | Yes | Yes | Yes | No | No | No | Yes |

*Abbreviations: CS: circular stapled, HS: hand-sewn, LSSM: linear stapler/semi-mechanical, OHS: One-layer hand-sewn, DLHS: Double-layer hand-sewn, TLHS: Three-layer hand-sewn, TS: triangulating Stapled*

**Table S4** Summary of other intraoperative and postoperative outcomes included in network meta-analysis

|  |  | **Operating Time** |  |  | **Blood Loss** |  |  | **Cardiac Complications** |  |
| --- | --- | --- | --- | --- | --- | --- | --- | --- | --- |
|  | **Studies, n** | **OR (CI_95%_)** | **p-value** | **Studies, n** | **OR (CI_95%_)** | **p-value** | **Studies, n** | **OR (CI_95%_)** | **p-value** |
| **All Studies** |  |  |  |  |  |  |  |  |  |
| CS *versus* HS | 5 | -41.86 [-149.22; 65.50] | 0.453 | 11 | -14.26 [-25.41; -3.11] | **0.012** | 7 | 1.02 [0.75; 1.40] | 0.909 |
| LSSM *versus* CS | 0 | -82.50 [ -253.96; 88.97] | 0.351 | 0 | -9.70 [-33.36; 13.97] | 0.43 | 0 | 0.74 (0.33, 1.67) | 0.469 |
| CS *versus* TS | 1 | 30.00 [-229.16; 289.16] | 0.832 | 1 | -8.00 [-49.61; 33.61] | 0.719 | 1 | 1.28 [0.37; 4.51] | 0.712 |
| LSSM *versus* HS | 4 | -124.35 [-258.05; 9.34] | 0.068 | 4 | -23.95 [-44.83; -3.08] | **0.024** | 2 | 0.75 (0.35, 1.59) | 0.465 |
| HS *versus* TS | 0 | 71.86 [-208.66; 352.38] | 0.628 | 0 | 6.26 [-36.82; 49.33] | 0.788 | 0 | 1.26 [0.34; 4.60] | 0.741 |
| LSSM *versus* TS | 0 | -52.50 [-363.25; 258.26] | 0.753 | 0 | -17.70 [-65.57; 30.17] | 0.478 | 0 | 0.95 [0.21; 4.24] | 0.951 |
| **Anastomosis Level** |  |  |  |  |  |  |  |  |  |
| **Cervical** |  |  |  |  |  |  |  |  |  |
| CS *versus* HS | 2 | -36.36 [-213.70; 140.98] | 0.701 | 3 | -18.83 [-39.12; 1.45] | 0.068 | 3 | 1.23 [0.71; 2.12] | 0.467 |
| LSSM *versus* CS | 0 | -103.59 [-339.37; 132.19] | 0.396 | 0 | -8.07 [-37.79; 21.64] | 0.607 | 0 | 0.56 (0.2, 1.56) | 0.273 |
| CS *versus* TS | 1 | 30.00 [-252.43; 312.43] | 0.846 | 1 | -8.00 [-48.74; 32.74] | 0.713 | 1 | 1.28 [0.37; 4.51] | 0.712 |
| LSSM *versus* HS | 3 | -139.95 [-295.33; 15.42] | 0.077 | 3 | -26.91 [-48.62; -5.19] | **0.015** | 1 | 0.68 (0.28, 1.64) | 0.405 |
| HS *versus* TS | 0 | 66.36 [-267.13; 399.85] | 0.71 | 0 | 10.83 [-34.67; 56.34] | 0.654 | 0 | 1.05 [0.27; 4.11] | 0.949 |
| LSSM *versus* TS | 0 | -73.59 [-441.50; 294.32] | 0.708 | 0 | -16.07 [-66.50; 34.35] | 0.543 | 0 | 0.72 [0.14; 3.65] | 0.706 |
| ***Thoracic*** |  |  |  |  |  |  |  |  |  |
| CS *versus* HS | - | - | - | 1 | -6.00 [-12.72; 0.72] | 0.08 | - | - | - |
| LSSM *versus* CS | - | - | - | - | - | - | - | - | - |
| CS *versus* TS | - | - | - | - | - | - | - | - | - |
| LSSM *versus* HS | - | - | - | - | - | - | - | - | - |
| HS *versus* TS | - | - | - | - | - | - | - | - | - |
| LSSM *versus* TS | - | - | - | - | - | - | - | - | - |
|  |  | **Pulmonary Complications** |  |  | **30-day Mortality** |  |  | **In-hospital Mortality** |  |
|  | Studies, n | **OR (CI_95%_)** | **p-value** | Studies, n | **OR (CI_95%_)** | **p-value** | Studies, n | **OR (CI_95%_)** | **p-value** |
| **All Studies** |  |  |  |  |  |  |  |  |  |
| CS *versus* HS | 8 | 1.15 [0.89; 1.48] | 0.285 | 7 | 1.80 [0.98; 3.31] | 0.058 | 16 | 2.16 [1.36; 3.44] | **0.001** |
| LSSM *versus* CS | 0 | 0.66 (0.35, 1.23) | 0.198 | 0 | 0.18 (0.06, 0.54) | **0.002** | 5 | 0.15 (0.08, 0.28) | **<0.001** |
| CS *versus* TS | 2 | 2.03 [0.74; 5.57] | 0.17 | 1 | 1.99 [0.08; 50.32] | 0.689 | 3 | 0.99 [0.34; 2.87] | 0.987 |
| LSSM *versus* HS | 2 | 0.76 (0.43, 1.35) | 0.348 | 3 | 0.33 (0.13, 0.81) | **0.016** | 14 | 0.32 (0.19, 0.54) | **<0.001** |
| HS *versus* TS | 0 | 1.77 [0.63; 5.00] | 0.284 | 0 | 1.11 [0.04; 29.61] | 0.955 | 2 | 0.46 [0.16; 1.35] | 0.154 |
| LSSM *versus* TS | 0 | 1.34 [0.41; 4.40] | 0.642 | 0 | 0.36 [0.01; 11.00] | 0.579 | 1 | 0.15 [0.05; 0.46] | **0.001** |
| **Anastomosis Level** |  |  |  |  |  |  |  |  |  |
| **Cervical** |  |  |  |  |  |  |  |  |  |
| CS *versus* HS | 3 | 1.55 [0.85; 2.83] | 0.154 | 3 | 1.37 [0.44; 4.23] | 0.598 | 3 | 1.05 [0.42; 2.62] | 0.924 |
| LSSM *versus* CS | 0 | 0.44 (0.19, 1.02) | 0.056 | 0 | 0.45 (0.05, 4.35) | 0.497 | 1 | 1.04 (0.34, 3.23) | 0.948 |
| CS *versus* TS | 2 | 2.03 [0.74; 5.57] | 0.17 | 1 | 1.99 [0.07; 54.86] | 0.699 |  |  |  |
| LSSM *versus* HS | 1 | 0.68 (0.37, 1.23) | 0.206 | 2 | 0.61 (0.09, 4.35) | 0.637 | 4 | 1.1 (0.5, 2.44) | 0.826 |
| HS *versus* TS | 0 | 1.31 [0.41; 4.26] | 0.664 | 0 | 1.46 [0.04; 48.38] | 0.845 |  |  |  |
| LSSM *versus* TS | 0 | 0.89 [0.24; 3.32] | 0.872 | 0 | 0.89 [0.02; 49.11] | 0.958 |  |  |  |
| *Thoracic* |  |  |  |  |  |  |  |  |  |
| CS *versus* HS |  | - | - | - | - | - | - | - | - |
| LSSM *versus* CS |  | - | - | - | - | - | - | - | - |
| CS *versus* TS |  | - | - | - | - | - | - | - | - |
| LSSM *versus* HS |  | - | - | 1 | 0.27 (0.1, 0.76) | **0.013** | - | - | - |
| HS *versus* TS |  | - | - | - | - | - | - | - | - |
| LSSM *versus* TS |  | - | - | - | - | - | - | - | - |

*CS: circular stapler, HS: Hand-sewn, LSSM: linear stapler/semi-mechanical, OR: Odds ratio.*

**Table S5** Summary of outcomes in previously published meta-analyses

| Study Name | Study Year | Techniques Compared | Study Types | Number of Patients | Anastomotic Leaks,  OR (CI95%) | Anastomotic Strictures,  OR (CI95%) | Mortality,  OR (CI95%) | Operative Time,  min (Range) |
| --- | --- | --- | --- | --- | --- | --- | --- | --- |
| Kim*^11^ | 2010 | HS vs CS | 8 RCTs | 710 | NR | NR | NR | NR |
| Markar^14^ | 2011 | HS** vs Stapled | 9 RCTs | 762 | 1.06 (0.62 - 1.80) | 1.76 (1.09 - 2.86) | 1.71 (0.82 - 3.56) | -2 (-3 - 0) |
| Honda^10^ | 2013 | HS** vs CS | 12 RCTs | 1407 | 1.02 (0.66 - 1.59) | 1.67 (1.16 - 2.42) | 1.64 (0.95 - 2.83) | -15 (-28 - -2) |
| Wang^13^ | 2013 | HS** vs CS | 9 RCTs | 871 | 1.30 (0.87 - 1.92) | 0.97 (0.47 - 1.99) | 0.83 (0.43 - 1.58) | 18 (-3 - 39) |
| Liu^12^ | 2014 | HS** vs Stapled | 15 RCTs | 2337 | 0.77 (0.57 - 1.04) | 1.45 (1.11 - 1.91) | 1.52 (0.97 - 2.40) | NR |

*Abbreviations: CS: circular stapled, CI95%: 95% confidence intervals, HS: hand-sewn, NR: not reported, OR: Odds ratio, RCTs: Randomised controlled trials*

* this study did not report meta-analysis

** referent group
